# Adaptation of a Hearing Voices Group Facilitation Training for VA Stakeholders

**DOI:** 10.1007/s10597-022-00975-1

**Published:** 2022-05-16

**Authors:** Erica Hua Fletcher, Ippolytos Kalofonos

**Affiliations:** 1 UCLA/VA Center of Excellence for Veteran Resilience and Recovery, UCLA Jane and Terry Semel Institute for Neuroscience and Human Behavior, 760 Westwood Plaza, Los Angeles, CA 90095-1759 USA; 2 Desert Pacific Mental Illness Research, Education, and Clinical Center (MIRECC), VA Greater Los Angeles Healthcare System, 11301 Wilshire Boulevard, Building 218, Los Angeles, CA 90073 USA; 3 Health Services Research & Development (HSR&D), Center for the Study of Healthcare Innovation Implementation & Policy, VA Greater Los Angeles Healthcare System Health Services, 11301 Wilshire Boulevard, Los Angeles, CA 90073 USA; 4 UCLA Center for Social Medicine and Humanities, UCLA Jane and Terry Semel Institute for Neuroscience and Human Behavior, 760 Westwood Plaza B7-435, CA 90095-1759 Los Angeles, USA

**Keywords:** Hearing voices, Psychosis, Peer support, Quality improvement, Group facilitation, Training evaluation

## Abstract

The Hearing Voices (HV) Movement promotes diverse understandings of voice-hearing and seeing visions, which mental health professionals commonly refer to as ‘auditory hallucinations,’ ‘schizophrenia,’ or ‘psychosis.’ Central to this movement are peer support groups through which attendees connect with others who have similar experiences. This paper describes an adaptation of a Hearing Voices group facilitation training at VA Greater Los Angeles (VAGLA) and discusses training modifications, along with trainee perceptions and implementation and intervention outcomes. This is a first step towards adapting HV-inspired groups to VA systems of care. Data collection involved surveys of trainees (n = 18) and field notes throughout the 24 h online training. Findings indicate high acceptability and appropriateness of the training and high feasibility in implementation, suggesting the training was well-adapted to VAGLA. This research contributes to global efforts to integrate the Hearing Voices approach in diverse settings and increase awareness about its benefits among providers.

## Introduction

The Hearing Voices (HV) movement is a global movement that promotes diverse understandings and approaches to navigate hearing voices, seeing visions, and other unusual and/or extreme experiences which mental health professionals commonly refer to as ‘auditory hallucinations,’ ‘schizophrenia,’ or ‘psychosis’ (Jones et al., 2016; Corstens et al., 2014; Escher & Romme, 2012). The HV movement developed out of a partnership between social psychiatrist Marius Romme, his voice-hearing patient Patsy Hague, and researcher Sandra Escher in the Netherlands (Romme & Escher, 1989). Since the late 1980s, the movement has spread to over 30 countries, including the United States. It is an international collaboration between professionals, people with lived experience, and their families to practice an alternative approach to coping with emotional distress in a way that is empowering and useful to people and does not start with the assumption that they have a chronic mental illness.

Central to this movement are peer support groups, often led by two facilitators who encourage attendees to discuss their unusual experiences and make meaning from them (HVN-USA Charter, 2020). Many groups affiliated with Hearing Voices Network-USA (HVN-USA) are facilitated by peers with lived experience who have completed the HVN-USA training, while some groups include pairs of a clinician and a voice-hearer. Facilitators uphold a common set of values that emphasize accepting and making sense of unusual experiences, an openness to all explanatory frameworks (biomedical, psychological, spiritual, paranormal, etc.), sharing diverse pathways to coping and recovery, and exploring the role of psychosocial adversity in the onset and persistence of unusual experiences (Longden et al., 2018). Unlike more structured self-help approaches, such as Alcoholics Anonymous or Wellness Recovery Action Plan groups, HV groups do not follow a standardized format, and regional networks have developed unique approaches tailored to their own specific contexts (Jones et al., 2016). They often draw on the clinical tools and approaches developed and disseminated through the broader HV movement, including the Maastricht Interview, voice dialogue, and experience-focused counseling (Romme & Morris, 2013; Schnackenberg & Martin, 2014; Longden et al., 2021). Attendees see HV groups as a place to normalize and reframe their understanding and form a more neutral or positive relationship to their unusual experiences (Hornstein et al., 2020). HV groups have been shown to improve social connectedness and self-acceptance (Meddings et al., 2006; Oakland & Berry, 2015; Dos Santos & Beavan, 2015; Beavan et al., 2017). Findings from a recent observational survey of 113 HV group participants in the U.S. reported, “helpful changes in their voices, their relationships, and/or how they understood their voices as a result of attending the group (Hornstein et al., 2020, p. 7).” A U.K.-based quantitative observational study by Longden et al., (2018) found that HV group participants (n = 101) reported improvements to their emotional wellbeing and social relationships, and also documented an improvement in clinical scores in association with participation.

Most HV groups are organized in community spaces, outside of clinical settings and health systems. This is partly due to the fact that the groups grew out of a social movement that advocates for the reform of mental health service provision and prioritizes personal experience as a key source of evidence, while maintaining a critical perspective of traditional medical research methods (Corstens et al., 2014). At the same time, an empirical research base has emerged, such as that cited above, along with discussions of whether and how to bring the benefits of HV groups into patient-involved research and mainstream systems of care (McCarthy-Jones, 2017, pp.314–316; Styron et al., 2017). Some advocates argue that “clinicians and systems of care might benefit from learning from the HVN [Hearing Voices Network] and from adapting the way unusual experiences are viewed, conceptualized, and ‘treated’,” but emphasize the need to do so in partnership with service-users in ways that maintain the user-led, self-help principles of the HVN (Styron et al., 2017, p. 780). Additional challenges to the integration of HV groups within mental health systems involve the great diversity and variability in how facilitators are trained and how groups are run. A nationwide survey of U.S. HV group facilitators (n = 32) found that all surveyed believed facilitators should have some training, while half believed the training should be standardized. (Jones, 2016; p.111–112). These findings indicate that additional research is needed to adapt HV groups to and train HV group facilitators within a variety of institutional contexts while maintaining the ethos of similar training conducted globally, as led by voice-hearers in alliance with clinicians (Longden et al., 2013).

This paper evaluates an adaptation of a HVN-USA group facilitation training, as a part of a larger pilot study to adapt and implement HV-inspired groups at the VA of Greater Los Angeles (VAGLA). The VA is a healthcare system that serves a diverse population of Veterans and one that is well-positioned to scale such groups: It supports robust peer support specialist training and employment opportunities, including peer-facilitated support groups (Klee et al., 2019; Chinman et al., 2012). It offers a range of services for Veterans with serious mental illness diagnoses, including, housing support, intensive case management, and recovery and rehabilitative services. The VA is also a site of innovative research, and VA stakeholders - including providers and Veterans - are accustomed to contributing to studies to improve care. One goal of the training was to expose clinicians to the HV movement values and approaches, thus fostering collaboration and greater understanding among mental health professionals, peer support specialists, and Veterans with unusual experiences.

To our knowledge, the HVN-USA group facilitation training has only been delivered twice at two VA centers nationally, and this is the first to include a quality improvement evaluation component. This evaluation used qualitative survey data and fieldwork to evaluate an HVN-USA group facilitation training with VA stakeholders. This paper aims to describe training adaptations, reasons for such adaptations, and implementation and intervention outcomes. This information will enable us to tailor future training to meet the needs of VA stakeholders at VAGLA. Moreover, this study can support the adaptation of HV-inspired groups in a range of community mental health settings.

### Intervention

The HVN-USA facilitator training consists of the following components: 24 h of interactive instruction by trainers with lived experience of unusual mental states, a 250-page facilitators’ workbook, and several short homework assignments. Table 1 compares the standard delivery of this training to adaptations made for VAGLA training. Trainees are given role-playing scenarios to practice facilitation skills and values, including curiosity, non-judgment, and respect for diverse ways people understand their voices. Following their successful completion of the course, trainees receive a training certificate and access to a national monthly call to support HV facilitators across the country. The training is typically held in person over 3 days for 8 h each day; however, due to the COVID-19 pandemic, the training was held over video conference (Zoom) in January 2021, in 4 h blocks twice a week for 3 weeks. Table 2 provides curriculum content for each of the 6 training sessions.

**Table 1:** Comparison of the HVN-USA facilitator training model to the VAGLA adaptation

Typical U.S. HV group facilitation training	VA adaptation
In-person, in a community setting	Virtual (due to COVID-19)
Goal of having equal numbers of voice-hearers and non-voice hearers/clinicians	Majority non-voice hearers/clinicians
Civilian trainers	1 civilian trainer & 2 Veteran trainers
Role-playing scenarios focus on issues commonly faced by civilians	Role-playing scenarios tailored to Veterans’ issues

**Table 2:** HVN-USA facilitator curriculum at VAGLA

Theoretical framework (intervention)	Romme and Escher’s approach (1993) to accepting, analyzing, and making meaning from unusual sensory experiences led to the development of an international network of support groups to build community among voice hearers.
Trainer characteristics	A nationally-recognized civilian voice-hearer who has been affiliated with HVN-USA for an extensive period; a U.S. Army Veteran social worker-in-training who experiences extreme states and who has served multiple tours in Afghanistan; a U.S. Air Force Veteran voice-hearer with experience in peer support facilitation education and patient-centered research.
Structure of intervention	24 h of formal training (the equivalent of 3 working days). 6 4 h video conferencing sessions conducted twice a week for 3 weeks, with a 1 h makeup session for those who were unable to attend a portion of the training. Short homework assignments
Training Content	Combination of didactic teaching and experiential group exercises, such as role playing and mock group participation and facilitation. Attitudes and beliefs about unusual sensory experiences assessed before and after training.
Session 1	Group introductions Training agreements & attendance policy Group exercise on recognizing clinical language and using lay language in the training Exercise on beliefs about unusual experiences Veteran trainer’s recovery story
Session 2	History of the HV Movement Present the evidence-base about the connection between trauma and unusual experiences Civilian trainer’s recovery story Discussion on challenging aspects of psychiatric involvement (social/self-stigma, involuntary commitment, psychotropic medication side effects, etc. )
Session 3	Stages of Recovery Voice-mapping & voice dialogue Coping strategies for unusual experiences HV approach: validation, curiosity, vulnerability, community Role-playing scenarios Discussion about self-advocacy with health care providers
Session 4	HVN-USA Charter, 2020 Affiliated vs. full groups, family & friend groups Discussion on the feasibility of starting groups at VAGLA HV group structure Role of the group facilitator Navigating challenging group dynamics
Session 5	Multiple role-playing scenarios Discussion on self-disclosure among VA clinicians
Session 6	Group exercise co-facilitating a HV group Planning HV groups at VAGLA
Session 7 (1 h makeup)	Multiple role-playing scenarios

### Conceptual Framework

This evaluation is guided by a retrospective application of Model for Adaptation Design and Impact (MADI), a comprehensive framework to understand the impact of adaptations and their relationship to intervention and service outcomes (Kirk et al., 2020). MADI conceptualizes relationships among three domains: (1) adaptation characteristics (e.g. modifications, nature of adaptation, adaptation decision-making, target intervention group, and timing), (2) mediating and moderating factors, and (3) implementation outcomes (e.g. adoption, acceptability, appropriateness, feasibility, cost, penetration, fidelity, sustainability) and intervention outcomes (e.g. client outcomes and service outcomes). Likewise, both adaptation characteristics and mediating factors affect the impact of the intervention. Table 3 applies the MADI to training adaptations made at VAGLA.

**Table 3:** Model for adaptation design and impact applied to HV facilitator training at VAGLA

Adaptation Characteristics	Moderating Factors	Outcomes
**Modification** Training Delivery **Nature of the adaptation** Tailor training elements, including anecdotes & role-playing scenarios in the curriculum), for VA stakeholders. **Adaptation decision-making** HVN-USA facilitators determined training delivery adaptations, via consultation with the research team prior to the training. **Target Audience** VA stakeholders enrolled in the HVN-USA training **When Adaptations Occurred** Adaptations occurred systematically and proactively pre-implementation.	**Goal for Adaptation** To tailor training elements and increase relevance and fit for VA stakeholders. **Systematic** Training delivery adaptations were made via a consultation process with VA stakeholders. **Proactive** Adaptations were made due to anticipated challenges, prior to the start of the intervention.	**Implementation** *Acceptability* Participants were highly receptive to the training, and many noted its utility in building new skills and developing alternative frameworks to understand unusual sensory experiences. *Appropriateness* Participants noted the potential benefit of HV groups among Veteran voice hearers and believed they could support self-advocacy and community integration. *Feasibility* Participants noted low barriers to starting HV groups at VAGLA, and technical questions about billing and charting were resolved prior to the close of the training.
		**Intervention Outcomes** *Trainee Outcomes* 16 out of 18 trainees completed the course and received certification. *Service Outcomes* 29 Veterans participated in 5 pilot HV groups facilitated by 10 trainees.

## Methods

This evaluation was led by a social researcher (EHF) and psychiatrist/medical anthropologist (IAK). The larger pilot study was assessed and funded in part by the UCLA Center of Excellence for Veteran Resilience and Recovery, with consultation and approval from a Veterans Engagement Group (VEG) (Fletcher et al. 2022). The VEG consisted of several Veterans voice-hearers, many of whom had also experienced homelessness. This group met several times to discuss the study. Given that the HV movement is peer-led and champions voice-hearers as integral to research about HV groups, we recognize that this evaluation did not include significant input from Veterans with lived experience of intense mental states. As researchers allied with HV values, we seek to create future opportunities for the co-production of research with Veteran voice-hearers, but are also limited by our potential biases as non-voice-hearers and current structural barriers to co-production at the VA. These challenges are described in the “Discussion” section, the implications of which are discussed in the “Limitations” section.

### Study Design

We used mixed methods to study training adaptations, via pre and post-training surveys and exploratory fieldwork at the training. Both surveys took between 15 and 45 min to complete and contained semi-structured questions and close-ended questions, based on the research aims. They were adapted from standard VA training evaluations and the HVN-USA training material. The pre-training survey was conducted via phone interviews or online throughout the week before the training, and it included general questions about trainees’ roles and responsibilities at the VA, their interests in the training, and goals for attending the training. The post-training survey solicited information regarding the acceptability, appropriateness, and feasibility of the training, as well as recommendations for further adaptations to tailor the training to VAGLA. It included the following questions: What feedback do you have about your experience in the Hearing Voices training? How do you think Veterans may respond to participating in a Hearing Voices group? What are some challenges or barriers you might face as a prospective Hearing Voices facilitator in the VA? The post-training survey was conducted within two weeks after the close of the training.

### Participant Recruitment and Characteristics

Trainees were recruited via convenience sampling, as is common for pilot testing. Recruitment methods included word-of-mouth, announcements at VA staff meetings, and talks associated with UCLA/VAGLA, as a part of efforts to inform VA administrators and staff about the larger study to adapt and implement HV-inspired groups within the VA. Additional outreach efforts were made to recruit an equal number of Veteran voice-hearers and clinicians, a goal for all HVN-USA facilitator training sessions. Recruitment for the evaluation began following enrollment confirmation. Trainees (n = 18) were VA researchers (including the co-authors), mental health providers, and students (n = 11); Veteran peer support specialists (n = 4); and Veteran service users (n = 3). The cohort included 5 self-identified voice-hearers, according to the pre-training survey, while many others mentioned infrequent unusual sensations, such as experiences of voice-hearing, throughout the training. Table 4 lists demographic information. Participants did not receive any compensation for their engagement in the evaluation.

**Table 4:** Demographics of HV Facilitation Trainees at VAGLA

Total	Number	Percentage
Gender		
Male	11	61
Female	7	39
Race		
White	8	44
Black	5	28
Latinx	3	17
Mixed Race	2	11
Education		
Graduate degrees/in progress	11	61
Undergraduate degree or some college education	7	39
Role		
Clinician	5	28
Peer support specialist	5	28
Voice-hearer/VA service user	2	11
VA affiliates (researchers, students, clinicians-in-training)	6	33
Self-identification		
Voice hearer	5	28
Non-voice hearer	13	72

### Interviews and Fieldwork

Surveys were administered over the telephone or online. In total, we collected 15 pre-training surveys and 14 post-training surveys. EHF conducted surveys over the phone and entered participant responses verbatim into a digital platform. In addition, EHF and IAK - health services researchers with experience in ethnography - took independent, detailed field notes throughout the 24 h training, and EHF took field notes on the 1-hour makeup session with 3 trainees and one trainer. A focused ethnographic approach enabled us to explore complexities associated with training implementation and gather in-depth, multimodal information about trainees’ perspectives and their participation in the training (Knoblauch, 2005). Fieldnotes were taken during and immediately after each training session, following a debrief with trainers. Researchers also met weekly to discuss data collection, any issues that arose during the training, and emergent themes from group conversations.

### Data Analysis

Survey data and field notes were loaded into qualitative analysis software (Atlas.ti 9), and EHF reviewed the data set to develop preliminary codes and create a preliminary coding framework and code definitions, based on MADI. EHF and IAK discussed codes and their definitions, from which we developed a codebook. We coded the full data set independently to ensure all relevant findings were represented, then met to discuss any discrepancies between our coding. We then identified themes and subthemes, organizing them hierarchically. To apply MADI retrospectively, we first described adaptations using domain 1 (i.e. adaptation characteristics), selected and measured relevant outcomes from domain 3 (i.e. acceptability, appropriateness, and feasibility), and indicated reasons for adaptations and how outcomes were achieved from constructs in domain 2 (Kirk et al., 2020, p. 11). To strengthen our analysis, 4 VAGLA trainees and 2 HV trainers engaged in member-checking processes to confirm that evaluation results were aligned with their perspectives on the training. This served to increase the accuracy of our findings (Flick, 2007).

The authors report no potential conflicts of interest with respect to research, authorship, and/or publication of this article. All authors certify their responsibility for the manuscript. This project was formally approved as a quality improvement project by VAGLA Institutional Review Board Administration and by the UCLA Institutional Review Board Administration.

## Results

Major themes include (1) adaptation characteristics and (2) implementation outcomes, with implementation sub-themes describing the acceptability, appropriateness, and feasibility of the intervention. The subtheme of acceptability is discussed further with respect to codes related to its defining characteristics: using lay language, active learning, and practicing vulnerability.

### Adaptation Characteristics and Moderating Factors

Training adaptations involved the inclusion of 2 Veteran trainers with lived experience; their use of personal anecdotes about military culture, deployment, re-entry to civilian life, and mental health and substance use challenges; role-playing exercises based on Veteran-specific scenarios; and continuous dialogue between VA researchers and trainers throughout the training. These adaptations were intended to increase the acceptability and appropriateness of the training. In addition, the participation of VA researchers leading the larger pilot study allowed for questions about the feasibility of implementing core features of HV-inspired groups at VAGLA to be answered before the close of the training.

Modifications to the existing facilitator training occurred in the planning phase and pilot implementation and were made by external trainers contracted through HVN-USA, via consultation with the evaluation team. The decision to include two Veterans on the team of three trainers was proactive, while minor changes to trainers’ approach to engage VA stakeholders were made in response to feedback from the investigators throughout the training. These included modeling active participation and soliciting responses from trainees throughout the course. Other systematic, proactive decisions made prior to the intervention included the modification of specific training elements to address common challenges faced by Veteran voice-hearers; these informal modifications to the training delivery included Veteran-related anecdotes throughout the training and tailored role-playing scenarios. These adaptations were intended to increase the relevance and fit of the training for VA stakeholders.

### Implementation Outcomes

Salient quotes about the acceptability, appropriateness, and feasibility of the training are listed in Table 5.

**Table 5:** Quotes about implementation outcomes

Acceptability	“This training contributes to me changing my approach to clients to allow them to determine how they want to interpret their experiences.” “It is amazing to see how much openness there can be among people…I’ve found it to be really powerful, to listen to the deep wisdom in the room working through uncomfortable things.”
Appropriateness	“Thinking back to the training, there seemed to be a lot of interest, a lot of excitement. It appeared that Veterans could be interested in having a space that isn’t a clinical space, a treatment space, and if they knew that other Veterans who heard voices are leading them… It [the training] did give me hope that this could be valuable to people, like it seemed valuable to some of the participants in the [training].” “I think there would be a great response, because I know a lot of Veterans who want to get help, who want to better themselves and become a better person. Veterans - we have a sense of community and brotherhood, so that is included in it [a HV group] as well.”
Feasibility	“We work with Veterans who hear Voices. We work with the Vietnam Vets, the Iraq and Afghanistan Vets. The team I’m on, we work with [homeless-experienced] Vets. There are plenty of opportunities to start Hearing Voices groups in our team.” “I pretty much do all the [peer support] groups… I pay attention to the consensus [and needs] around me to decide what kind of groups I want to do. I am in control of everything I do up here.”

#### Acceptability

Trainees indicated that the training intervention was satisfactory, engaging, and useful, as well as an interesting contrast to clinical approaches. In the pre-training survey, stakeholders expressed the following reasons for their enrollment: to learn about the experience of having unusual mental states, to share diverse cultural frameworks for interpreting mental illness beyond biopsychiatric categories, to build skills working with Veteran voice-hearers, to increase their effectiveness as clinicians, to support Veterans and their family members, and to be a part of bringing a promising intervention to VAGLA. One trainee noted, “As a [clinician-in-training], I use [psychiatric] language to describe client experiences. . But in my culture and within my family, we are all driven by something, a presence in our mind and spirit that moves us. Spirits or ancestors are guiding me. I try to honor them.” Following the training, several trainees noted that they had a greater appreciation for the lived experience of voice-hearing, lay language to discuss unusual mental states, and multiple frameworks to interpret unusual sensations and beliefs.

##### Using Lay Language

Instructors indicated throughout the training that all frameworks for understanding unusual sensations or beliefs are embraced in HV groups; however, as an exercise in moving beyond biopsychiatric frameworks, they pointed out instances when trainees used clinical language about mental health throughout the course and advised them to use lay descriptors of what they felt or experienced instead. For example, ‘auditory hallucinations’ and ‘paranoia’ could be replaced with ‘hearing voices’ and ‘feeling as though you are being watched.’ This practice served to remind trainees that HV groups aim to make spaces for people to explore and discuss their experiences in alternative ways and find balance within many frameworks. Interestingly, Veteran voice-hearers in the group strongly identified with their psychiatric diagnoses and their roles as patients; they found it difficult to understand reasons to use lay language for mental distress. Reflecting on the training, one Veteran stated, “ I wasn’t fully on board with changing names - throwing out psychiatric diagnoses. [My doctors] are my providers - I don’t mind them talking about me like I was a patient.” Veterans’ reactions to this pedagogical strategy are understandable in the context of their socialization into military culture, adherence to medical hierarchies, psychoeducation from clinicians about their mental illness, overall positive regard for the VA, and lack of formal mental health training. Moreover, their psychiatric diagnoses, in part, enabled them to access VA services and related benefits, which many found to be crucial in their recovery.

Expressing some hesitancy about moving away from clinical frameworks, a clinician-in-training asked the trainers, “I wonder how newly-experiencing [diagnosed] voice-hearers adapt to this model? I wonder if the [psychiatric] diagnosis can be a container. Sometimes you need a polarity [between non-clinical and clinical approaches] to figure out the balance for yourself,” to which the Veteran trainer affirmed that he needed a scientific framework of mental illness initially, to become more open to spiritual frameworks and to group support. These discussions about the utility of clinical language, as well as their unintended effects, helped trainees become more attuned to their language choices and aware of their motivations for using such terms. In contrast, some clinician stakeholders noted the sense of freedom they felt in using lay language to talk about mental distress and engaging with Veterans as “experts by experience.” One mental health provider explained,

The longer I do my job, the more I learn that diagnoses are theories, and our knowledge. is not finished or complete. . I like allowing everyone to have their own way of thinking. and not having to use the terminology, the symptoms, the medical model… It gave me. more space and time. I’d like to give that to my patients. It always feels rushed when I am. doing an intake, trying to understand what their symptoms are; I think it [HV-inspired groups] would. be a good opportunity to have patients talk about these things without the psychoeducational component, having them come up with their coping strategies, instead of telling them all the time.

A Veteran peer support specialist similarly appreciated the ability to “let people just be, not try to change things.” Another clinician remarked, “This is a more process-oriented group. For me, it’s a breath of fresh air. In psychoeducation, I do most of the work and the talking. It’s good to step back and let people talk about their experiences and not jump to challenge, to explain, or to change [them].” Overall, the trainers’ emphasis of lay language aimed to convey their desire to counterbalance the dominant framework of biopsychiatry with alternative epistemologies and the role of group facilitators to welcome all perspectives with curiosity and care.

##### Active Learning

Trainees noted that these role-playing scenarios and group facilitation exercises were quite memorable and pedagogically useful to demonstrate the HV approach and help them understand their roles as future group facilitators. Instructors enacted the following scenarios based on common Veterans’ experiences: a Veteran who felt unsupported by the government and family while reintegrating into civil society; a Veteran whose friend who died in combat and returned to her as a voice encouraging her to complete suicide; a Veteran who sought emotional support from VA providers, but was met with a brisk discussion of psychotropic medication compliance and risk management. In response, trainees would practice validating the emotions expressed, asking questions to learn more about the situation, telling similar stories and experiences from their own lives, and reassuring the Veteran that they are not alone. Some clinicians felt that the role-playing scenario allowed them to form “a human connection” with Veterans, “without having the provider hat on.” A VA-affiliated student noted, “My favorite part of the training was when we did the role-playing [exercises]. . It was challenging to let go of the problem-solving orientation, and just the impulse towards fixing something. . I think it was challenging, and it was a moment that I felt like I was learning what the approach was really about, which was not that.” Of the role-playing scenarios, a Veteran voice-hearer noted, “I really thought what was being said was [geared] towards Veterans, and the little groups - the breakout rooms - were geared towards getting information from the Vets about how they feel- what [they are] experiencing.”

##### Practicing Vulnerability

At the beginning of the training, clinicians struggled with sharing their own stories and emotions - what trainers termed ‘practicing vulnerability’ - to better relate to struggles faced by Veteran voice-hearers. At times, Veteran voice-hearers and peer support specialists wondered if they were sharing too much of their own life experiences. Some mentioned that they felt intimidated by the clinicians in the training session or taken aback by their reticence to open up with the group. One peer remarked, “The clinicians - they kind of just sit back and even though they aren’t saying nothing, they are, like, diagnosing; and the peers are like, ‘What, what, what?’ Because we are curious. And the Vets are like, ‘Wow, we can share this?’ The ones who don’t say anything…. That left a bad taste in my mouth.” Similarly, some trainees did not fully understand the importance of sharing personal stories and emotions within group role-play exercises, as a way to model one of the key components of HV facilitation and to build a sense of community within a group space. One participant remarked, “I was. . unsure about the confessional nature of the training. The training began with personal narratives from the facilitators, but at times the lines between training and personal disclosure [blurred].” This sentiment indicates an unease felt by some VA employees when asked to listen carefully to trainers’ stories of struggle, survival, and recovery and to adopt a more relational understanding of professionalism among colleagues and Veterans.

Over time, clinicians became more comfortable discussing their reticence practicing vulnerability, and one mentioned that they felt unsure about how and when to practice forms of self-disclosure. Others also felt that their struggles felt less important to bring to the group, given that many Veterans had mentioned adversities such as homelessness, incarceration, and substance use challenges. The group discussed that a part of professionalism -- in the context of group facilitation -- included mentioning similar feelings to validate what Veteran voice-hearers may share and that they should contribute what felt appropriate to them as a clinician. Towards the end of the training, clinicians felt more at ease implementing validation strategies that drew from their life experiences. In turn, trainers explained that clinicians did not need to have the same lived experience of unusual mental states as Veterans, to relate to similar feelings or emotions. For example, in response to a Veteran voice-hearer’s description of relationship issues, one clinician mentioned that they had recently gone through a breakup and similarly had to take inventory of what they wanted in a relationship and partner. In another instance, one trainee noted that seeing how another trainee responded to a role play -- by sharing about a time in their life that was similarly difficult to a Veteran in crisis -- helped them recognize the therapeutic aspects of validation and self-disclosure. These small, but meaningful gestures served to create a sense of greater group cohesion and momentarily destabilized traditional roles held by professionals and patients/service users.

#### Appropriateness

Trainees indicated perceived relevance of HV-inspired groups among Veteran voice-hearers and believed they would benefit from participation in the intervention. Perceived benefits included an opportunity for connection among Veterans; peer education about coping strategies; a nonjudgmental space to discuss spirituality, unconventional beliefs, and other sensitive subjects; acceptance of unusual experiences; and prevention from cycles of psychotropic medication withdrawal, homelessness, and incarceration. A peer specialist stated, “Even virtual camaraderie and virtual companionship [would be welcomed]. . I’m noticing a lot of guys are gobbling up any opportunity just to hear a friendly voice during these trying times.”

Perceived barriers to Veteran engagement included self-stigma and a lack of knowledge about the approach. Describing Veteran service users, a peer specialist noted, “The individuals I deal with are in denial. . How do I convince them to come to a meeting? They don’t want to be classified like that [as a voice-hearer].” In addition, concerns about the level of “functioning” needed for a Veteran to participate in and benefit from a HV-inspired group remained with at least one trainee/clinician following the training. The participant noted, “Here is my concern - there is a part of me that feels like if [Veterans] hear voices, they need to be stable. Are they honestly in a situation where they can operate an iPad and be beneficial to someone else? When [a Veteran] is super medicated, he is not functional on the same [level].” This comment revealed a challenge some clinicians had in attempting to move away from pathologizing frameworks for categorizing the “severity” of a serious mental illness, despite assurances from the trainers that the HV approach could be used in any context -- including hospital and forensic settings -- with people currently experiencing intense and unusual mental states.

#### Feasibility

Trainees indicated that implementing HV-inspired groups in VAGLA was highly feasible. Veteran peer support specialists (hereafter referred to as peer specialists) reported great autonomy over their ability to start and facilitate support groups. One peer specialist stated, “ I could set it [a HV-inspired group], and I could do it virtually right now. I would have their [administrators’] blessing. . The clinicians [would] refer the Veterans hearing voices to me.” VA clinicians also noted that they were well-resourced and able to start a group as soon as recruitment had been completed, given that their supervisors were already aware of the training and supportive of their roles as future group facilitators. Concerns about the feasibility of starting groups largely centered around technical challenges, such as teaching VA providers, peers, and Veterans how to join a virtual meeting and addressing digital access issues. A peer specialist noted, “There are some [Veterans] who we’re going to have to set up. . There is going to be a logistical challenge if they have a smartphone, [or] if they [need] technical support. . That is a consideration for me.” Technical questions about digital access as well as charting, coding, and billing were resolved by the end of the training^1^: trainees were advised to write general notes about themes brought up during groups, and a standard note title for the adapted HV groups was created in the VA electronic medical record.^2^ Given the VA’s funding structure, billing issues that would warrant substantial consideration in a U.S. county mental health system did not need to be addressed at this stage of implementation.

### Intervention Outcomes

#### Stakeholder Outcomes

Sixteen out of 18 trainees completed the course and received certification as HV group facilitators. Twelve out of the 14 evaluation participants who completed post-training surveys agreed or strongly agreed with the statement, “As a result of this training, I feel better equipped overall to work with Veterans who hear voices and experience altered mental states,” while two marked this statement as neutral. After completing the training, one peer specialist noted,

The overall approach of the training left you feeling that you get to see a different point of view. I’ve never seen the point of view from someone who hears voices. I just heard that point of view, and I’m more cognizant of it, and I’m going to be more caring, more understanding of that point of view. I now have more value for their experience.

Additional skills trainees noted from the training include supporting Veterans’ self-determination of their experiences, creating a stronger therapeutic alliance between clinicians and Veterans, and using nonclinical language to discuss unusual sensory experiences and mental distress.

A VA employee and a Veteran voice-hearer did not complete the training; they named a lack of interest and difficulty engaging in 4 h, biweekly video conference calls/concentrating on topics discussed respectively as reasons for deciding to leave early.

#### Service Outcomes

Of the 16 trainees who completed the training, 10 went on to lead 5 adapted HV groups, called ‘Veteran Voices & Visions (VVV) groups’ at VAGLA. As a part of the larger study, each group was closed (limited to service-connected Veterans, whom research team members screened for eligibility and provided informed consent). Groups were organized by cohorts of Veterans with similar ages (and one group of women Veterans of all ages), co-facilitated by at least one VA mental health care provider and a VA peer support specialist, and recorded for later observation.^3^ These weekly, virtual groups supported 29 Veteran voice-hearers, and following the 4-month study period, all but one group has continued to meet (as of January 2022). Bi-monthly meetings with researchers and VVV facilitators enabled the continued education of facilitators, including discussions about group progression, HV values, HV resources to share with Veterans, and reflections on collaborative processes between VA clinicians and peer support specialists.

## Discussion

The Hearing Voices movement, as a global alliance of voice-hearers, family members, researchers, and clinicians, creates critical community spaces for people to discuss unusual experiences and beliefs without fear of censure or judgment (Longden et al., 2013). This approach has the potential to normalize highly stigmatized experiences of “psychosis” among Veterans and support their sense of belonging and social integration (Wastler et al., 2020). In turn, bringing the HV approach to the VA aligns well with the VA’s strategic priorities to provide patient-centered care, improve health care access via technology, offer greater choices, and support Veterans’ whole health (Department of Veteran Affairs 2019). This article presents ways to adapt a facilitator training in the VA context, along with potential strategies for further adaptations in future implementation efforts. Given many HV activists’ uneasy relationship with traditional research methods, challenges associated with establishing an evidence-base for HV groups, and the need for evidence to scale HV groups in mental health care systems, we aim to advance initial steps to implement the HV approach in the VA, as a part of larger shifts within HV research towards establishing a robust evidence-base (Dillion & Hornstein, 2013; Corstens et al., 2014; Styron et al., 2017; Longden et al., 2018).

MADI offers a conceptual framework for retrospective analysis of adaptation characteristics that affect implementation and intervention outcomes. Analyzing the training through this lens enabled us to identify strengths and challenges to the training’s acceptability and appropriateness among stakeholders, as well as its perceived feasibility. Via MADI, we identified VAGLA-specific adaptations to improve the practice adaptations and described participant perceptions about the training. The training adaptations listed in this article are intended to be descriptive, not prescriptive to other VA centers. Indeed, the adaptations made at VAGLA and the way in which the training came about was highly situational, based on site-specific factors, such as stakeholder interest in patient-led approaches to improve community integration, grant funding, and pre-existing relationships between investigators and HVN-USA leaders. Findings suggest adaptations of the HVN-USA facilitator training were responsive to the needs of VA stakeholders; the high retention rate of trainees (89%) in the time-intensive training suggests their continued interest and the training’s relevance to meet the needs of Veteran voice-hearers. VA stakeholders also perceived high feasibility in running HV-inspired groups at VAGLA, and the majority of trainees (n = 10, 56%) went on to facilitate such groups. Applications of these findings can improve the relevance of future facilitator training.

A challenge to integrating the HV approach in the VAGLA context included the adoption of a non-clinical framework and language to describe intense or unusual sensations and experiences. Veteran voice-hearers initially had some difficulty understanding reasons for using lay language to describe unusual mental states and felt hesitancy considering themselves in ways beyond their traditional roles as patients or service users at the VA. Meanwhile, many clinicians found it difficult to practice self-disclosure to relate to other group members in co-facilitated HV group role-play scenarios, while peer specialists and Veteran voice-hearers found sharing their lived experience to be an affirming aspect of the training. Such challenges were noted as key features of the training’s high acceptability and appropriateness among participants, and they served to foster a sense of collaboration and mutual learning within the group that destabilized aspects of traditional patient/provider hierarchies and other interprofessional hierarchies at VAGLA. Trainees reported feeling a sense of freedom to interact with Veterans and other VA stakeholders in a less structured, prescriptive manner.

### Potential Strategies to Improve Training Adaptation and Integration

VA stakeholder engagement in planning meetings with HVN-USA trainers and in the research process could have improved the relevance and acceptability of both (Wendleton et al., 2019). For example, the failure to retain one Veteran voice-hearer over the course of the training could indicate a need to create additional accommodations to ensure Veteran engagement. Following the training, many participants also suggested the recruitment of a more equal ratio of Veteran voice-hearers to clinicians in the cohort to prevent the tokenization of Veterans and increase the representation of diverse perspectives.

Future efforts to implement a HV group facilitation training in this context may benefit from the creation of a curriculum tailored to Veterans’ diverse needs and the VA context (including training manual and lesson plans) (See Horstein et al., 2021). Given some challenging issues Veterans raised in pilot VVV groups, potential topics for additional facilitation training may include: managing hostility between group participants, racial stereotypes/race relations issues, bullying by military supervisors/peers, sexual/domestic violence in the military, self-harm, and incarceration histories. In addition, the creation of fidelity measures for running HV-inspired groups could enable a better evaluation of a trainee’s performance and assess facilitators’ adherence to the model.^4^ Some participants noted that the length and content of the training could be streamlined, to enable greater stakeholder access and participation. Education among VA staff and administrators about the HV approach could increase awareness and support of the HV-inspired groups, while infrastructure to provide continuing education and support for facilitators could help ensure program sustainability. The employment of VA stakeholders as trainers, especially Veteran voice-hearers, could increase the acceptability, appropriateness, and reach of the training. Continued research on the integration of the HV approach in diverse health care systems could contribute to a greater understanding of regional adaptation strategies to tailor existing HV facilitator curricula to specific populations and settings.

### Limitations

Study limitations include an observational design and survey data collected from a small convenience sample of VA stakeholders within a single VA health care system. Exploratory findings should not be generalized outside of VAGLA or the USA. In addition, VA staff had collegial relationships with the research team, which could have impacted their generally positive responses. To our knowledge, this is the first study to evaluate a HVN-USA facilitator training, and because of this, we do not have comparisons to assess intervention and implementation outcomes.

With regard to stakeholder representation, the recruitment of Veteran voice-hearers to the facilitation training was limited by (1) the small number of Veteran voice-hearers (3 Veterans) involved in a pilot HV group at VAGLA prior to this project’s start, (2) a limited pool of VA-employed peer support specialists who identified as voice hearers or other unusual experiences, (3) and an inability to compensate non-VA employees (i.e. Veteran voice-hearers) for training as facilitators and for facilitating pilot VVV groups. Furthermore, current mechanisms to solicit stakeholder engagement in research processes (such as the VEG) are typically one-off events, without long-term mechanisms to sustain (e.g. fund) and train Veterans as research consultants and/or members of VA research teams (Fletcher et al. 2022). The co-production of research with Veteran stakeholders could have enabled a more nuanced approach to and diverse perspectives about the evaluation of the facilitator training, while adhering to HV values for inclusivity of voice-hearers in research, education, and group facilitation (Jones et al., 2016; Longden et al., 2018; Hornstein et al., 2020). These barriers reproduce epistemic inequalities within knowledge generation and have the potential to lead to the co-optation of the HV movement within a system of integrated care that is known for its top-down implementation of peer support specialists services, rather than its ability to generate grassroots and internal support for local and nationwide peer-led initiatives (Chinman et al., 2012; See also Jones at al., 2020).

Lessons learned from this evaluation and the larger pilot study have the potential to increase program relevance for specific populations and settings within the VA and beyond. However, in our own work, we are also concerned about the possible co-optation, mainstreaming, and neutralization of the most unique aspects of HV groups and participatory processes via more traditional methods, should future program implementation and implementation research at the VA be limited to peers, clinicians, and researchers without lived experience (Crichton et al., 2017; Faulkner, 2017; LeBlanc & Kinsella, 2016). Indeed, because we seek to produce research that is representative of and responsive to the needs of Veterans, we have reflected on the promises and perils of bringing HV-inspired approaches to the VA, given current constraints. That being said, we are also wary of overvaluing folk politics (i.e. grassroots organizing over systemic transformation) or identity politics (e.g. stances that only voice-hearers should participate in research about HV groups) as bellwethers for “good” mental health social movement strategies for system transformation (Jones & Kelly, 2015; Kelly, 2016; Kalathil & Jones, 2016; Longden et al., 2018). Given increased interest in HV groups and their implementation in forensic, clinical, and community organizations in recent years, we maintain that this research supports a burgeoning evidence base that could have major implications for systems of care interested in adopting meaningful elements of the HV approach. Therefore, we aim to take current opportunities to pilot HV approaches in the VA to contribute meaningful strategies in practice adaptation, while planning to incorporate Veterans, HV leaders, and HV researchers as researchers and consultants in future research and implementation initiatives.

Identifying adaptations made to a HV facilitator training and their impact within a national health care system has the capacity to enhance future training, implementation, and sustainment efforts. These findings contribute to emerging scholarship on the spread of the HV movement globally and within specific systems of care, as a part of larger efforts to implement recovery-oriented approaches and patient-led initiatives (Hornstein et al., 2020; Steel et al., 2020). Preliminary research indicates that HV groups can support mental wellbeing via peer support and community integration in a wide range of health care and community settings; however, more research – including robust participatory research – is needed to determine the appropriate content and length of facilitator training and facilitator characteristics suitable to support HV group participants (Jones et al., 2016).

## Abbreviations

HV: Hearing Voices
HVN-USA: Hearing Voices Network - USA
MADI: Model for Adaptation Design and Impact
VAGLA: Veteran Affairs of Greater Los Angeles
VVV: Veteran Voices & Visions

## Footnotes

1 The VA has a program providing a tablet to facilitate remote access for Veterans who do not already own a device with a screen/video capacity.

2 Specific questions about liability were also addressed; however, that topic is outside the scope of this paper.

3 A thorough description of adaptations made within our Veteran Voices & Visions groups is outside the scope of this paper, however, we plan to publish a separate article on this topic in the coming months.

4 Plans to develop a facilitation manual and fidelity measure are underway. Both will draw our analysis of pilot VVV group dynamics, along with guidance from HV movement leaders and VA stakeholders.
